# Navigating competing needs: a qualitative study on parenthood with a partner with Huntington’s disease

**DOI:** 10.1080/21642850.2025.2465614

**Published:** 2025-02-17

**Authors:** Kristin J. Billaud Feragen, Sidsel Egedal, Siri Hagen Kjolaas

**Affiliations:** Centre for Rare Disorders, Division of Paediatric and Adolescent Medicine, Oslo University Hospital HF, Oslo, Norway

**Keywords:** Huntington’s disease, partners, parents, family, children

## Abstract

**Objective:**

Huntington’s disease (HD) is a rare neurodegenerative condition characterised by progressive symptoms affecting motricity, cognition, neuropsychiatric function and behaviour. HD develops during a period of life in which many live in partnership and have children. HD impacts all family members through its cognitive and psychological symptoms, mid-life onset, long disease trajectory and genetic risk. The aim of the study was to explore how parents without HD experience and manage parenthood when their partner is affected by HD.

**Methods:**

Qualitative interviews with 14 caregivers were analysed using reflexive thematic analysis.

**Results:**

Three main themes with corresponding subthemes were identified, followed by an underlying theme: *Genetic risk: An underlying layer of complexity.* The first theme, *Balancing competing demands,* describes the challenges involved when attempting to attend to conflicting needs within the family*.* Theme 2, *Needing a shoulder to lean on*, covers participants’ feelings of loneliness and their need to be seen by others, whereas Theme 3, *Restoring and building strength*, encompasses coping strategies used by caregivers to protect themselves and their children from potential negative experiences. The underlying theme describes how the genetic aspect of the disease permeates the participants’ experiences across all other themes.

**Conclusion:**

Support providers may be unaware of the extensive repercussions HD can have on a family. Acknowledging the central role of partners without HD and their risk of psychological distress is crucial. Exhausted partners may struggle to support their children, which may lead to childhoods overshadowed by HD. For family members to prioritise their own needs, tailored support must be set in place for parents with HD.

## Introduction

Huntington’s disease (HD) is a rare autosomal dominant and neurodegenerative condition characterised by motor disturbances as well as cognitive, psychological/neuropsychiatric and behavioural changes (Stoker et al., 2022). On average, HD results in death 10–20 years after onset (Ghosh & Tabrizi, 2018). HD is caused by a trinucleotide repeat (CAG) expansion in exon 1 of the *HTT* gene, which is located on chromosome 4 (Nance, 2017). Individuals are diagnosed based on the display of motor symptoms (Novak & Tabrizi, 2010; Stoker et al., 2022), with a mean age of onset of 30–50 years (Roos, 2010), a period of life in which many are in committed partnerships and take on shared responsibilities for children.

HD has a profound impact on those who develop the disease, with elevated levels of stress, depression, anxiety, psychosocial distress, and reduced quality of life (Dale et al., 2022; Dale & van Duijn, 2015; Epping et al., 2013; Kachian et al., 2019; van Lonkhuizen et al., 2023). HD also affects entire families and how partners work together as parents due to several of its symptoms: cognitive rigidity, mood changes, loss of organisational skills and changes in personality and behaviour (Aubeeluck et al., 2012). Partners may end up holding primary caretaking responsibilities for both the affected person and the children at the same time (Karlstedt et al., 2021). Research in this area points to disrupted family dynamics; adverse experiences; difficult emotions such as worry, guilt and depression; and reduced social participation and support (Aubeeluck et al., 2012; Decruyenaere et al., 2004; Kjoelaas et al., 2020; Kjoelaas et al., 2022a; Williams et al., 2012; Wittenberg & Prosser, 2013). HD’s onset in the middle of one’s life, a long disease trajectory, and the complexity of having children who are at genetic risk of contracting HD are additional challenges (Etchegary & Fowler, 2008; O’Connor et al., 2008; Wibawa et al., 2020). Nevertheless, persons with HD have also reported satisfaction with family life (Jona et al., 2017), and research has shown that coping strategies and social support can alleviate the negative impact of the disease on caregivers (Karlstedt et al., 2021; Palacio et al., 2020).

Complex challenges that extend far beyond HD’s physical symptoms may be present years before a clinical diagnosis, and partners and children may be among the first to notice and struggle with the psychological and behavioural changes that come with the disease (Achenbach & Saft, 2021; Considine et al., 2023; Kjoelaas et al., 2020; Kjoelaas et al., 2022b; Leidl et al., 2023; Youssov et al., 2022). The impact of HD on the whole family has also been summarised in a recent review (Cooper et al., 2024), highlighting some of the complex challenges children face when growing up with a parent affected by HD, and the possible effects of the disease on attachment and social relationships.

A qualitative review on partners of individuals affected by a motor neurone disease (Holkham & Soundy, 2018), another neurologic condition whose symptoms include the progressive loss of motricity, summarised the disease’s main challenges as follows: a loss of control, a lack of choice (obligation to care), social isolation and a change in relationship dynamics. HD and other degenerative diseases come with the painful processes of losing what had once been reciprocal physical and emotional spousal support and ‘falling out of love’ (Decruyenaere et al., 2004; Williams et al., 2009). The partner with HD may, on the other hand, not experience the same loss in partnership quality (Reininghaus et al., 2012), which may be partly explained by their lack of self-awareness of deficits (anosognosia), which is estimated to affect 25–50% of individuals with HD (Wibawa et al., 2020). Anosognosia may lead to higher levels of caregiver burden and reduce the shared understanding and approach to parental roles and priorities (Petzke et al., 2022; Williams et al., 2009).

In summary, HD impacts the whole family. Although some research has focused on partners’ experiences, few studies have explicitly investigated how partners deal with their dual caregiving role. Therefore, the aim of this study was to explore the lived experiences of partners of a parent affected by HD, primarily focusing on caregiving experiences.

## Method

A qualitative approach using semi-structured interviews was chosen to address this work’s broad research question: What are the experiences of caregivers without HD when navigating parenthood alongside a partner with HD?

### Participants

Caregivers who currently have children or had children who are now adults and have a partner with HD were invited to participate. To facilitate purposive snowball sampling, information on the study and invitations to participate were distributed through various channels, such as educational courses on HD, genetic counselling services at Oslo University Hospital, Haukeland University Hospital (Bergen), and St Olav Hospital (Trondheim), the Norwegian Association for Huntington’s Disease, and relevant websites. The provided information sheets detailed the study’s objectives and main interview topics and included the researchers’ contact details. Saturation was not a criterion during recruitment, and all participants who contacted the research team for participation were included, as long as they fulfilled the inclusion criteria. Fourteen individuals provided written consent and agreed to participate. The participants’ mean age was 54.9 years (range 42–69 years). Additional demographic information is provided in Table 1.

**Table 1.** Demographic characteristics of participants (*N* = 14).

Characteristics		
Children’s age group	School-aged children (0–12 years)	3
	Teenagers (13–18 years)	4
	Young adults (19–35 years)	5
	Adults (36–45 years)	2
Participant gender	Female	11
	Male	3
Level of education	Secondary education	4
	Higher education	10
Marital status (in relation to a partner with HD)	Widowed	4
	Married/partnership	8
	Divorced	2
	Widowed	4

Participants are labelled ‘parent without HD’ in the following sections.

Family constellations varied, ranging from current to past caregiving and partnership experiences. The interviewer did not ask about the children’s genetic status; however, three participants shared that their children were born without risk of inheriting HD.

### Procedure

The data were part of a larger study that specifically investigated the experiences of children who grew up in families affected by HD (Kjoelaas et al., 2022a, 2022b, 2022c). The data revealed that partners experienced unique challenges. Therefore, the present study exclusively focuses on topics from the interviews conducted with parents without HD and their experiences with being a caregiver when their partner has HD – a facet of the data not comprehensively addressed within the scope of the larger study.

Guides for interviews with parents without HD were developed based on the research literature on HD, feedback from three clinical experts and inputs from user representatives. The clinical experts consulted for this study consisted of three counsellors with extensive experience working with families affected by HD. The user representatives were three individuals with first-hand experiences of either growing up in a family with HD or being a partner/parent of someone with HD. The three user representatives were members of the larger study’s reference group and were involved in all phases of the study.

### Measures

Participants provided demographic information (see Table 1 for details) prior to participating in a semi-structured interview. The interviews, conducted individually in a semi-structured format, investigated both challenges and protective factors (see Table 2 for interview topics and sample questions). The semi-structured interview format made it possible to adapt the conversation and explore topics participants expressed as meaningful to them.

**Table 2.** Main topics and sample items from the semi-structured interview guide.

Interview topic	Sample questions for caregivers without HD
Background information	*Could you please describe the family your children grew up in?*
Childhood experiences	*How did your children experience the disease? How do you think growing up with a parent with HD affected your children?*
Disease- and self-disclosure	*How did your children first learn about HD? What do you feel are the benefits and disadvantages of disclosing information regarding HD to children? What information and conversations about HD did you feel like your children needed?*
Resources and support	*What sources of support did your children have growing up? What support could your children have needed that they may not have received?*

### Interviews

Interviews were carried out by the last author, a PhD candidate at the time of the study, who was formally trained to address sensitive topics owing a master’s degree in clinical and health psychology. The first author, an experienced licenced clinical psychologist and senior researcher, participated in the first two interviews to provide feedback on interview skills. With the exception of two participants whom the last author had previously met at a meeting for the Norwegian Association for Huntington’s Disease, the author had no prior relationship with any of the participants. Interviews (July 2019–August 2020) lasted 60 minutes on average (range: 27–90 minutes). All interviews were conducted face-to-face with the exception of one, which was done via telephone. Interviews were carried out at the Centre for Rare Disorders at Oslo University Hospital and at various external locations, including the participants’ homes. The interview location was determined by the participants, with most choosing the privacy of our outpatient department due to challenging home environments.

### Data analysis

This study is guided by a phenomenological framework and aims to describe subjective perspectives and participant’s processes of meaning-making (Teherani et al., 2015). Braun and Clarke’s reflexive thematic analysis approach was used (Braun & Clarke, 2006; Braun & Clarke, 2019). The interviews were recorded and transcribed verbatim. In the initial two steps of the analysis, the first and last authors carefully read and reread the transcripts. Next, the first author generated initial codes by isolating phrases, sentences or paragraphs to form a comprehensive list of codes across transcripts. The code list was grouped under broader categories that described similar phenomena and discussed between all three authors. The themes were reviewed against the data and were discussed until a consensus was reached regarding their content and structure before the final themes were selected and named. The report was then written, presenting our participants’ experiences (analytic narrative), with participants’ quotes to illustrate some of the findings. Pseudonyms have been used to ensure anonymity. All main points within the Results are supported by data (participant accounts).

### Reflexivity and trustworthiness

Reflexivity was emphasised throughout the analysis. The first and last authors drew on prior experience with Huntington’s disease (HD) as researchers in their analysis. To enhance the research’s validity and mitigate potential researcher bias, the first and last author formed part of the primary analytic team so that the second author, who had extensive clinical expertise as a genetic counsellor but no prior research experience with families with HD, could serve as a discussant. This approach allowed the authors to leverage their diverse perspectives and critically examine their theoretical and professional interpretations. Consensus was achieved through multiple rounds of independent readings, code – and note-sharing, open discussions and iteratively rereading and rediscussing the interviews.

### Ethical considerations

The study received ethical approval from the Regional Committee for Medical Research Ethics [Health Region East, Norway, reference number: 2017/1613]. The participants were informed about the study, including their confidentiality and right to withdraw at any point, in adherence to the principles outlined in the Declaration of Helsinki. Given the sensitivity of the topics discussed in the interviews, arrangements were made for relevant referrals or follow-ups, if needed.

## Results

**Figure 1.**
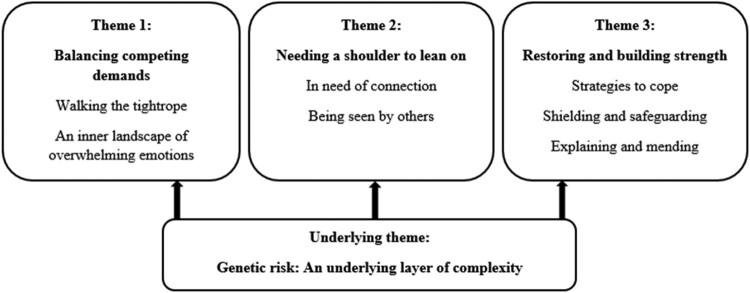
Thematic map.

The analysis resulted in three main themes, with corresponding subthemes, and an underlying theme, Genetic risk: An underlying layer of complexity*,* that describes how the genetic aspect of the disease permeates the participants’ experiences across all three main themes (see Figure 1 for an overview of themes and subthemes). The first theme, *Balancing competing demands,* includes two subthemes, *Walking the tightrope,* and *An inner landscape of overwhelming emotions,* describes the profound emotional repercussions of the participants’ attempts to navigate competing needs. Theme 2, *Needing a shoulder to lean on*, includes the participants’ descriptions of loneliness and their need to be seen by others, whereas Theme 3, *Restoring and building strength*, covers coping strategies caregivers used to protect themselves and their children from the potential negative effects of the disease.

Single quotation marks are used when formulations are the participants’. Square brackets [ ] are used to indicate clarifications, such as when participants refer to previous information or name someone. Parentheses (…) indicate that an extract has been removed for editorial concision.

## Theme 1: Balancing competing needs

### Walking the tightrope

Although participant experiences varied depending on disease phases and individual differences, navigating parenthood with a partner affected by HD was described as a multifaceted and complex balancing act. While the participants wanted to take care of their partner’s needs (thereby demonstrating loyalty), they also wanted to protect their children from potential adverse experiences (thereby exercising parental responsibility). The challenge was that these needs were increasingly in conflict and in some cases incompatible:

I have heard many people describe it as balancing on that knife’s edge. And that’s probably a very correct description, because you try in a crazy way … you spend a lot of effort to balance that existence. (Brian, father of young adults)

One participant said she felt ‘like an octopus’, trying to use all her tentacles to respond to conflicting practical and emotional needs. Participants felt pulled in different directions – akin to ‘doing the splits’ or walking on a tightrope – while trying to facilitate and organise daily life. Despite reshuffling priorities and reducing social activities to a minimum, everyday life was described as being ‘stretched to the limit’:

This is what I feel is so extremely difficult – that you are constantly doing the splits. And that you constantly feel you are insufficient in both ways. That you are never enough of a carer for the person who is ill and never a good enough mother. (Amy, mother of teenagers).

The urgency of contradictory needs varied depending on the situation and setting. In some cases, conflicting needs concerned daily choices, such as when to eat or how to spend an evening together. In other cases, the situation could be much more demanding or extreme, such as when dealing with a parent with HD’s threats or attempts to take his/her own life while also having to shield and support a potentially traumatised or scared child.

He was angry and said, ‘I’m going out to drown myself.’ I knowingly decided to stay with the children – stayed with them at home; I didn’t go after him. But I had my family on standby … so my [family] went looking for him. (Sarah, mother of school-age children)

Competing needs could lead to conflicts within the family. Partners seemed to avoid or diminish conflicts when they occurred rather than adopt longer-term solutions that could lead to an escalation of conflict. For example, they could allow a parent with HD to drive with the child on board (a short-term solution that would avoid a conflict about driving skills) instead of questioning the affected person’s ability to drive.

She should definitely not drive. (…) Nothing has happened yet, but [our child] has told me she will not sit in her [mothers’] car again.(…) She hasn’t told her that she won’t. But there will come a day [when we have to]. (…). She drove her last summer, and I was really tense. I hoped she would be okay. (…). And [daughter] said ‘Never again!’ (William, father of school age child)

In spite of challenges due to symptoms of HD, participants wanted to be loyal to the partner they had loved, chosen and/or had explicitly promised to take care of when they became ill. Knowing that the disease was responsible for the challenges they were facing was a strong incentive to remain loyal. Nevertheless, some felt that loyalty was not possible in the longer term if they perceived symptoms of HD had a negative impact on the child:

It’s really tough. You are married and have promised to stay together, but it just doesn’t work. (…). Because he was so changed as a person and only cared about himself and not the children. (Lucy, mother of young adults)

Some of the participants claimed that their feelings for their partner changed as a result of symptoms and daily challenges, which made it easier to prioritise their child’s needs. A parent with HD’s lack of insight regarding the changes that came with the disease was also experienced as altering partnership dynamics:

This is about empathy (…). When you get into a discussion or something like that (…), there’s no guilt, no regret, nothing – she’s not close to it (…), putting herself in someone else’s situation (…). If I had been mad at you, I would say, ‘Sorry!’ (…). She doesn’t say ‘sorry’. (…) And she used to care so much for the children. (John, father of teenagers and young adults)

Several participants said that their child’s well-being was a ‘non-negotiable priority’, even when it hurt to let down their partner, and indicated that they would probably have been more present for their children and taken better care of themselves if they had known that the parent with HD had been adequately taken care of. While trusting or knowing that their partner understood or accepted the need to take the progression of the disease into account helped the participants prioritise their children, it still generated feelings of guilt, as shared in the interviews:

It has been challenging; it is still challenging. For now, the children come first, [but I] feel guilty for not including him more [in family activities]. It feels strange not to include him. I feel uncomfortable about it. (Rachel, mother of school-age children)

In contrast, others said that they trusted that having HD in the family had not adversely affected their children, and one participant felt that his partner’s relationship with the children was good, even if the consequences of the disease had led the parents to divorce.

### An inner landscape of overwhelming emotions

Participants described being ‘on the brink of exhaustion’, ‘feeling inadequate and deficient’, compensating, never being sufficiently present and hoping to prevent their family from shattering. Participants felt emotionally overwhelmed by the situation, using descriptions such as ‘being at war’, ‘living in alertness’ and ‘constantly holding their breath’. For some, everyday life seemed to be going on as it would in the eye of a storm: external forces raged on the outside (overwhelming demands and incompatible needs) alongside a lack of awareness of how they dealt with the situation. Managing the chaos of everyday life was the primary focus, and they seemingly had little time or energy to stop, be mindful and present and assess the situation:

I have an enormous workload compared to what I had in the past. And with so little extra energy, this is about getting back on your feet and finding a new everyday life. This is what I focus on fully now. (…) Because I don’t have a spouse who calms things down; he just gears it all up. You know, I didn’t imagine this would be so hard. And I’m a really strong woman. I’ve experienced so much in my life, and I come out stronger from most experiences, and this is what I want here as well, but this requires everything I have. It requires hard work. (…) I stumble all the time, and I feel sad and miserable, I feel grief and … despair. And I’m angry, and there is so much going on at the same time, and it’s kind of chaotic to be right in the middle of all this. (Nicole, mother of school-age children)

Failing to fulfil everybody’s needs came with feelings of guilt: guilt if they felt they were letting their partner down and guilt for not being sufficiently attentive to their children’s needs. Some felt guilty about giving their child a parent on whom they could not rely on and/or were slowly losing. One of the participants described it as ‘a loss in waiting’, while others described sadness as episodic or something to be kept at arm’s length when one feels too vulnerable. Participants described a profound feeling of loss, sadness, grief and sorrow that pervaded and infused all aspects of daily life:

I said to my husband, I have a sadness inside me, or rather, I have many sorrows, but a great sadness that your life will not be like what you had hoped and dreamed of. I mourn that we are not an ordinary family. I have a sadness that you will die early. I am saddened that you will not see your children grow up. I am saddened that we will not become grandparents together or see our children (…) getting married. All these dreams we had, when we met and married and had children, have been so crushed. They vanish with this terrible disease. (…) It has been a grief that you must process, and it is a sorrow that the children will grow up, first with a father that is ill and then without a father. (Sarah, mother of school-age children)

## Theme 2: Needing a shoulder to lean on

### In need of connection

The participants’ experiences with the disease fostered a deep feeling of loneliness and lack of connection, both within and outside the family. Several aspects of loneliness were described: the progressive loss of their partner; the difficulty with sharing their reality with others; and the hardships involved in keeping and building social relationships because of the many challenges that came with the disease. All three aspects contributed to the participants’ feelings that they stood alone against HD.

Participants missed having someone who understood what they were going through and longed for the companionship they once had with their partner, with whom they could no longer discuss child rearing, daily disagreements and concerns, challenges, and life’s happy moments, and whose shoulder they missed being able to lean on when needed:

You lose your companion, who … you could have done things with together, such as raising children … (…) You have no one to lean on – sort of. In terms of things you wonder about, things you could have done together … yes. You become very alone. (Julie, mother of teenager and young adult)

The participants’ social relationships changed and grew complicated because of the disease, and some friends and social relationships were lost. Loneliness was also prompted by the feeling that nobody could really understand the reality of what they experienced. Other people’s complaints or stories about common everyday struggles, such as a partner’s forgetfulness or a fight with a teenager, were experienced as in sharp contrast to their own realities, intensifying a sense of solitude. Nevertheless, the challenges the family went through together could also ignite family cohesion.

### Being seen by others

Participants needed others to understand and see what their family was going through and recognise their needs for practical and emotional support.

Most participants had not experienced other people understanding their situation. Some, however, described how healthcare providers or others had given them a feeling of being seen and understood, and had expressed concern not only for the affected person, but also for the rest of the family, and/or acted upon this concern by asking the participants how they felt. Being seen could in itself be cathartic or the starting point of change that allowed emotional or practical support to be set in place.

Suddenly, [the health nurse] asked [the daughter]: ‘How are you?’ And she was completely taken aback because no one had asked her before. So she started to cry. That is the reaction she had when someone cared. (…). She asked again each time we met her, ‘Are you all right? Are you okay?’ And we were so grateful; someone saw us. (Mary, mother of teenagers)

Feeling seen by others helped participants assess whether they could or should recalibrate or adjust how they navigated choices and decisions on a daily basis. Other people’s input was important for those who struggled with where to set the boundaries. Feedback or comments could help the participants understand changes and behaviour and/or help them decide how to cope with opposing needs within their families:

I could see the children, how they reacted, become a bit like … , went to their room or crawled up in the chair or … . And I remember my father once saying, ‘This is not okay’. That something, something very unpredictable, comes into the room, and you don’t know what is going on. You just expect it to explode, but you don’t know when, and you don’t know why and you don’t know what you can do about it. (Amy, mother of teenagers)

Few participants had experienced external support from extended family, friends or healthcare providers. Consequently, some participants had not understood that something had to change or found the limit for what they and their families had to tolerate:

I needed my [psychologist] to help me cope with this. (…) I was exhausted, and I could see that my children were in so much pain (…) And then I thought, ‘I don’t know how I’m going to … I don’t know; I don’t understand how I’m going to cope with this anymore.’ By then, [psychologist] said to me, ‘Who can take the responsibility for you? (…) And who is responsible for your children?’ She never said to me, ‘You have to divorce.’ She never said that. In spite of following me up for many, many years (…). This was the moment when I realised that I had turned over all the stones so many times, and I was about to get sick myself. And my children suffer. (Lisa, mother of young adults)

These families’ need for support did not seem to have been identified, leading participants to struggle on their own. In some families, the parent with HD was the one hindering support by not recognising or accepting the need for it. Decisions on where to set the limits felt difficult, and doubt was a part of this equation. Therefore, feedback from the children, or expected or feared reactions from others, could be experienced by the participants as relief from having to deal with doubt on their own.

## Theme 3: Restoring and building strength

### Strategies to cope

Some participants described activities, conceptualisations or elements of daily life that helped them cope and recharge. One of the participants, for example, talked about working on ‘connecting her network to the family’ and building what she called a ‘support wall’. Her network ‘helped her build strength and recharge’, leaving her more present for her children.

Going to work and having what the participants called ‘a normal life’ was important to some, but was not possible for all, as they struggled with handling both work and responsibilities at home. One of the participants shared how she weighted ‘costs and gains’ and ‘kept only activities and friendships that helped her regain energy’ in her everyday life.

Several strategies were useful in terms of recharging: being mindful of small details, being present in daily life, taking one step at the time, being aware of keeping a balance between good and challenging life experiences, and keeping a special focus on gratefulness.

It’s really hard work. I think it’s … well, it’s (…), about [filling up with] joy, not necessarily big things. To be present here and now and appreciate the small things, whether it’s a good moment between [my husband] and the children or … like today, [he] actually went [to our daughter] and gave [her] a big hug before she went to school. (…) I write them down, these golden moments, in my gratitude book. (Nicole, mother of school-age children)

### Shielding and safeguarding

Participants attempted to protect and shield their children by, for example, finding places where they could stay after school if the situation at home was chaotic or by taking them away from the situation. A few participants described how finding another home for the parent with HD seemed to be the only solution. If a parent without HD feared that personality and behaviour changes negatively impacted their child, they would limit and monitor the child’s contact with the parent with HD. Some organised family activities that did not involve the parent with HD so that arising complications or feelings of shame would not cast shadows on the child’s experiences.

[Our last holiday together], everything just got messed up because he … well. He got lost, he fell and hurt himself; he was different than he usually was. And the children were embarrassed (…) and more and more stressed because they [feared embarrassment]. I don’t know … , but it’s kind of impossible to take him with [us when we do] things, because I have to think about the children – protect the children, somehow. (Rachel, mother of school-age children)

As an alternative to shielding their children, some families employed proactive measures aimed at strengthening positive experiences with the parent with HD. Examples of this include portraying the parent with HD in a positive way, facilitating joyful activities and showing pictures of or talking about the parent with HD before the disease changed them.

Participants tried to protect their children by suggesting how they could handle challenging situations and share useful strategies regarding what they could say or avoid saying if people had questions.

### Explaining and mending

Participants described their wish to explain, repair and mediate. For some, repairing had to be done on a daily basis. They explained to their children how the disease changed the parent with HD so that they would know that the harsh or mean words spoken to them were not what their parent really felt or meant:

I told [my daughter] that he was sick and that he didn’t mean [what he was saying]. And ‘you have to think that way, too. Deep inside, he loves you, and he doesn’t mean to hurt you’. (Mary, mother of teenagers)

Participants comforted their child when needed and emphasised that they were not responsible for the behaviours or feelings of their parent with HD. One participant described how her partner with HD, who was having an outburst, told his daughter that she was the worst thing that had happened in his life. The child also noticed changes in her father’s behaviour:

‘Mum, does dad really love me anymore?’ she asked. So, I said, ‘Why do you ask?’ ‘Because he doesn’t smile to me anymore, and we hardly do anything together anymore, like we did before.’ So, I needed to connect this [to what is happening], ‘This is Huntington’s disease. Dad loves you just as much as he used to, but it is harder for him to show it to you.’ (Nicole, mother of school-age children)

Talking about the disease and the changes that the children observed but did not understand, while also underlining the love that the parent with HD had for them, seemed to be the primary way of mending the potentially negative consequences of HD:

[The children] said (…), ‘Dad, what’s the matter with you? You act strangely nowadays’. So, I looked at him and said, ‘Now is the time’. So, we all sat down (…) and told them that Dad has an illness in his head and that the entire body’s engine is in the head. Sometimes, something goes wrong with the engine. (…) And sometimes there are spare parts, sometimes not. And for Dad’s illness, there are no spare parts … so Dad will get sicker, but he is very fond of you’. And those are the words they remember: ‘Dad is very fond of us’. (Sarah, mother of young adults)

Talking to the children about HD could be difficult if the parent with HD did not want information to be shared with other people or with their children, as they felt that there was no need to talk about symptoms that they did not see or perceive as problematic. This could be described as ‘an extremely difficult situation’ for the parent without HD:

For me, to [handle this] together, I needed to share the information (…). So one day, he came home from [work], and I was so distraught; he was so clearly sick, and it was so unbearable for my children (…). It took a year [for him to say] ‘We need to tell them’, and it was terrible. It was an extremely demanding period. (…) My [husband] sat there, and he provided some information, but he was saying ‘when I get sick’. (…) But I think the children understood. Because it made sense that Dad was sick, or that this was about an illness, right? (…) I also had conversations with them by myself. (Lisa, mother of young adults)

The disease could also introduce new perspectives into the family’s daily lives. Humour, such as laughing about unexpected and odd situations, apparently worked as a repairing and unifying element. Talking about the challenges experienced by the family also strengthened the connection between family members. Remembering family life before the appearance of symptoms seemingly had a therapeutic and repairing effect on the whole family.

One of the participants said that the parent with HD, during the later stages of the disease, wrote a song for their child as a message of love and as reparations for previous difficult years. Participants’ descriptions gave the impression that particularly powerful emotions were activated in participants when the parent with HD was the initiator of such reparations.

## Underlying theme: Genetic risk: an underlying layer of complexity

The added burden of genetic risk shaped parents’ perceptions, emotions and decision-making in profound ways, and was a fundamental underlying and complex issue across themes.

Having children with the knowledge that a parent possesses a hereditary disease was described as a complex challenge. One participant, for example, talked about how she struggled to cope with her own choices after terminating a pregnancy to ensure that her child would not develop HD. Another shared how other people’s concerns and judgments regarding her choice to have a child with a person at risk for HD were experienced as draining, and how she felt that she constantly had to explain her choice to other people.

Genetic risk was also experienced as a complicating factor when talking about the disease within the family. Although explaining HD was intended to protect the child and address potential negative impacts of the disease, participants found it deeply challenging or even impossible:

He knows that his mother is ill (…), that she gets tired and fusses and messes up things and all that it entails. But he doesn’t know anything more (…). [But he is] on the internet, and his friends are, or he may be with someone talking about it, so I fear that he might get some information that he doesn’t need – for example, information that he could inherit the disease. I would like to wait as long as possible for him to find out. (…). Learning that he could get the same disease as his mother is too great a burden for a child (William, father of school-aged children).

The uncertainty and/or knowledge surrounding their child’s genetic risk generated a deep sense of sorrow, guilt and worry that surpassed their sense of mourning on their partner’s behalf. Fear and ambivalence was described as casting shadows over occasions typically perceived as joyful by others, as exemplified by one participant who shared how the news of becoming a grandmother initially elicited more sadness than happiness, as she knew that there would be new generations at risk.

Genetic risk seemed to cause complex emotions to arise within the context of familial relationships. One of the participants shared how her teenage daughter blamed her for not checking whether she would develop HD before her birth. Another participant described how her decision-making was coloured by her wish to influence her children’s perception of a life affected by HD:

One of my mantras throughout this whole journey has been that we have to be with their father in a way that, if they become ill, they’ll think that he had a good life. (…) So that if, if, if (…) they will think that he had friends, that he went on vacations with his family, that he had people around him and that he had birthday parties with lots of people. (Amy, mother of teenagers)

## Discussion

Although previous literature has shed light on the impact of a neurodegenerative disease on family carers (Weitkamp et al., 2021; Wittenberg & Prosser, 2013), the specific symptoms and genetic nature of HD introduces distinct challenges carrying implications for healthcare and the provision of support. Partners without HD shared what seemed to be an ongoing and difficult balance between competing needs within the family when navigating parenthood with a partner with HD. Symptoms and changes that came with HD and their wish to be loyal to their partner appeared to compete with their children’s need for secure and stable relationships, a supporting home environment and emotionally available caregivers. Our study also sheds light on potential protective mechanisms and strategies for managing HD within the family.

## Coping with multiple and conflicting roles and demands

As with most parents, our participants took on multiple roles. While they were both partners and parents, they also had roles outside the family system (e.g. employees, friends and extended family members). As suggested by our results and by the literature, HD introduces a complicated interplay of physical, psychological and emotional challenges on top of more common parental roles and obligations, potentially affecting partners as well as their entire family (Wittenberg et al., 2013).

Role theory (Biddle, 1986) defines roles as a set of behaviours and activities that are expected from a person in a certain position, and how challenges may arise when roles appear to conflict with or oppose each other, leading to role overload. Our participants described experiences that could be interpreted as role overloads, role conflicts and role reversals. Research has indicated that navigating parenthood may be particularly difficult when symptoms of HD coincide with parental obligations for younger children (Domaradzki, 2015; Røthing et al., 2014), which may possibly be explained by higher levels of role conflict and/or role overload. Role reversals, such as romantic relationships built around care and dependency (Domaradzki, 2015), have also been described as emotionally demanding.

Research indicates that families affected by HD lose opportunities to engage in social activities and experience a lack of social support (Aubeeluck et al., 2012; Cooper et al., 2024; Decruyenaere et al., 2004; Røthing et al., 2014). The results from the present study may shed light on some of the processes involved in this phenomenon. The participants described navigating overwhelming emotional landscapes that were difficult to share with outsiders. They felt that no one could understand the reality of their daily lives, which fostered a deep feeling of loneliness. Hence, social interactions could reinforce feelings of isolation due to the contrast between two worlds (theirs and other people’s daily lives). This deep feeling of loneliness and struggle with sharing their own reality may potentially explain some of the mechanisms involved when the participants experience a lack of social support.

The interviews also indicate that when they felt overwhelmed, the participants tended to focus primarily on family members’ immediate needs instead of taking a step back and assessing their situation from a broader perspective. The participants’ ability to care for themselves seemed to have been lost along the way, as reported by previous studies (Aubeeluck et al., 2012; Parekh et al., 2018; Røthing et al., 2015). Our results indicate that the participants’ situation exceeded the available external and internal resources. Hence, theories of stress and coping (Lazarus & Folkman, 1984) could serve as adequate models for understanding caregiver burden in the context of HD. The theory of Lazarus and Folkman (1984) identifies two processes, cognitive appraisal and coping, as critical mediators of stressful situations and their immediate and long-range outcomes (Folkman et al., 1986). For cognitive appraisals to occur, an individual needs to evaluate whether a particular situation impacts their well-being and, if so, in what ways. This is followed by a secondary appraisal in which the person evaluates what, if anything, can be done to change the situation. For the participants who felt stretched to the limit and in the eye of the storm, this appraisal did not seem to be within reach unless someone outside the situation helped them evaluate and recalibrate their priorities. Research on other severe health conditions has indicated that a higher burden of care may lead to a greater adoption of negative coping strategies (Kazemi et al., 2021), highlighting the crucial role care providers have in identifying families in need for support and helping them evaluate their situation from an overall perspective and build adequate coping mechanisms.

## Loss of dyadic coping

The participants’ accounts, as well as research on HD, illustrate how symptoms of HD transform the relationship between the children’s two caregivers from a spousal and dyadic relationship into a caregiver relationship (Domaradzki, 2015; Etchegary & Fowler, 2008; Parekh et al., 2018). The participants gradually lost a shoulder to lean on and the partner they had hoped to collaborate with to rear children and live as a family. Complex changes are involved when a couple is affected by a severe disease. Partners may experience improved relationship quality but also struggle with demanding role shifts and caregiving responsibilities (Decruyenaere et al., 2004). Current and anticipated loss plays a major role in these relationships, spurred by the loss of the future individuals expect for the couple, their partner, and their children (Aubeeluck et al., 2012; Decruyenaere et al., 2004), as indicated in the present study. Other strong emotions were also described, including shame, guilt and blame, frustration and anger, tiredness and exhaustion, and distress and depression. Genetic risk was an additional burden that generated deep anxiety and sorrow. Notably, the quality and quantity of the provided support did not seem to fit the extent of support needed by the parent without HD. Few participants received help with processing strong and difficult emotions, assessing the child’s situation and recalibrating priorities, and adjusting their balance on a tightrope.

Dyadic coping theory (Bodenmann, 1995), building on Lazarus and Folkman’s stress theory (1984), specifically focuses on the dynamic interplay between romantic partners. One systematic review investigated the impact of severe chronic conditions on couples, how they communicate about stress and support each other, and how they deal with the consequences of disease (Weitkamp et al., 2021). The review identified communication about illness as a central factor in regulating the impact of disease on a family (Weitkamp et al., 2021). Dyadic coping theory (Bodenmann, 1995) and the findings from the aforementioned review (Weitkamp et al., 2021) suggest two core risk factors in families affected by HD. First, cognitive, psychological and behavioural symptoms, in combination with reduced social cognition (Ghosh & Tabrizi, 2018; Petzke et al., 2022; Williams et al., 2009) and anosognosia (Wibawa et al., 2020), may complicate a partner with HD’s ability to identify stress signals in family members and/or to manage dyadic coping, which would otherwise alleviate stress. Difficulties with recognising and processing negative emotions (Bora et al., 2016; Henley et al., 2012) will also limit the couple’s ability to communicate about stress and support each other when facing challenges. Second, communication about illness, identified as a crucial factor in regulating the impact of disease (Weitkamp et al., 2021), seems to be complicated by challenges involved in sharing information about HD (Cooper et al., 2024; Dalton et al., 2019; Kjoelaas et al., 2022b) and in some families by the individual with HD’s wish to withhold this information. Hence, specific symptoms of HD, as well as difficulties related to communication, may have direct consequences for partners’ ability to engage in dyadic coping.

## Scaffolding and mending

One of the most compelling reasons to ensure adequate support for parents without HD, beyond the need for self-care, is the role they play in safeguarding their children’s present and future mental well-being, which has been shown to be challenged in families affected by HD (Cooper et al., 2024; Daemen et al., 2024). When children face adversity, regardless of origin, their caregivers become pivotal resources in shaping their children’s coping mechanisms and adjustments. Adversity can impact the development of the brain and multiple body systems (Oh et al., 2018), and children who endure long-term distress face heightened risks of developing a range of mental and physical health issues (McCoy, 2013; McLaughlin et al., 2019; Thompson et al., 2015), as demonstrated in families with HD (Cooper et al., 2024; Kjoelaas et al., 2022a; van der Meer et al., 2012). In contrast, supportive relationships with trusted adults can buffer and shield children from the detrimental effects of stress and adversity in both the short and long term (Daemen et al., 2024; Kjoelaas et al., 2022a; Shonkoff et al., 2012). Psychological risk seems to be reduced when caregivers help the child to make sense of the situation and place the responsibility where it belongs (Kjoelaas et al., 2022a; Kjoelaas et al., 2022c). Caregivers can have conversations and encourage transparency (Cooper et al., 2024), which can help children develop an emotional understanding and coherent representations of past and current events (McCoy, 2013), as described by several participants in this study.

## Clinical implications

HD is a rare disease that the healthcare system and society in general lack knowledge of, as demonstrated in several studies across different countries (Domaradzki, 2016; Skirton et al., 2010; van Walsem et al., 2015; Zarotti et al., 2024). Our study suggests that providers of healthcare and other forms of support may be unaware of the tremendous impact and repercussions HD has on a family, particularly on partners and children.

Critical gaps in healthcare and social services have been identified in the follow-up of individuals with HD (van Walsem et al., 2015). Meeting the many care needs of a person with HD (Aubeeluck et al., 2012) is important so that their partners have more resources and time to take care of themselves and their children. Acknowledging the partner without HD’s central role and highlighting their risk of overwhelming psychological distress are crucial to avoid exhausted partners who may struggle to support their children, which in turn may lead to childhoods being overshadowed by the disease (Kjoelaas et al., 2022a). Hence, it is critical that patient-centred care adopts a family-centred approach (Mühlbäck et al., 2023).

Families affected by HD need help with strengthening social support and social participation, by evaluating family members’ subjective appraisals of the situation as well as their social and psychological resources (Daemen et al., 2024; Decruyenaere et al., 2004; Røthing et al., 2015; Williams et al., 2009). The family will also need guidance on increasing transparency by talking to their children about the disease, its consequences and its heritability (Cooper et al., 2024). Referrals for couple therapy or counselling that aim to strengthen dyadic coping and communication skills from early on in the disease progression (Petzke et al., 2022; Reininghaus et al., 2012) can be emphasised. However, additional research is needed on whether developed interventions benefit individuals with HD (Zarotti et al., 2020).

## Strengths and limitations

One major strength of the current study is its investigation of an insufficiently researched topic; few studies have explicitly and exclusively explored how parents experience and handle their role as primary caregivers when their partner is affected by HD. This study’s results may help researchers and healthcare professionals understand some of the many challenges that come with HD from a family perspective. Considering the scarcity of research on this topic, the study’s reflexive qualitative approach is another strength.

Some limitations must be highlighted. First, the participants were recruited through genetic counselling services and the National Association for HD. Parents who felt they were coping with their situation, had more personal resources and/or had reflected upon the child’s situation may have felt the study was more relevant for them. The opposite could also be true. Regardless, the interviews varied in terms of challenges that were shared. Furthermore, the participants provided an extensive range of lived experiences. Second, only three fathers participated. Future research should aim to include a higher proportion of fathers to mothers when exploring parental experiences. Third, participants were all living in (Country), which may restrict the results’ generalisability to other medical and social contexts.

## Conclusion

HD usually develops during a period of life when in which many live in partnership and exercise shared responsibilities over children. The present study improves our understanding of the impact of severe neurodegenerative diseases on a family – knowledge that should be used to improve interventions. The specificities of HD, combined with the many of the common challenges that come with this severe disease, have an emotional and psychosocial impact on all family members. Healthcare providers need to be aware of the extensive repercussions HD can have on the whole family and aim to identifying those in need of support. Acknowledging the partner without HD’s essential role in supporting the children of parents affected by the disease is crucial. Parents also require guidance on how to talk to their children about the disease and its consequences. Our study suggests that partners’ caregiving burden and conflicting roles deserve increased attention in research as well as in clinical follow-ups.

## Supplementary Material

SRQR_Checklist_.docx

## Data Availability

Data are not publicly available due to Oslo University Hospital’s privacy and ethical restrictions.
